# Retrospective practice review of treatment of metastatic non-small-cell lung cancer with second-line erlotinib

**DOI:** 10.3747/co.v15i6.382

**Published:** 2008-12

**Authors:** B. Melosky, J. Agulnik, H. Assi

**Affiliations:** * BC Cancer Agency, Vancouver, BC; † Jewish General Hospital, Montreal, QC; ‡ The Moncton Hospital, Moncton, NB

**Keywords:** Non-small-cell lung cancer, nsclc, epidermal growth factor receptor, egfr, tyrosine kinase inhibitor, tki, erlotinib, second-line, retrospective practice review

## Abstract

**Background:**

Epidermal growth factor receptor tyrosine kinase inhibitors (egfr-tkis) and chemotherapy have both demonstrated efficacy in recurrent metastatic non-small-cell lung cancer (nsclc) following failure of first-line platinum-based chemotherapy. Although the 3 available therapeutic agents—docetaxel, erlotinib, and pemetrexed—have significantly changed the treatment landscape for recurrent nsclc, the optimal selection of second- and third-line therapy has not been established. This practice review examines the outcomes in clinical practice of using second-line erlotinib followed by third-line chemotherapy in the treatment of recurrent metastatic nsclc.

**Methods:**

We conducted a retrospective review of nsclc patient charts at three Canadian institutions. Patients with recurrent nsclc who had received second-line erlotinib therapy followed by third-line chemotherapy were selected by census. A chart review assessed key outcomes that included time to progression (ttp), response, and change in performance status. Outcomes for specific patient subgroups were also examined.

**Results:**

We identified 35 patients for this retrospective practice review. First-line platinum-doublet therapy demonstrated a mean ttp of 6.6 months and a 46% overall response rate (15 partial responses and 1 complete response). Second-line treatment with erlotinib produced the highest mean ttp of all lines of therapy (9.2 months) and an overall response rate of 40% (all being partial responses). In the third-line setting, in which most patients received docetaxel, the mean ttp was 4.3 months and the overall response rate was 18% (all being partial responses). Subgroup analysis showed that all patient subgroups demonstrated benefit from second-line erlotinib treatment; improved benefit was observed in patients who developed rash, in female patients, in never smokers, in Asian patients, in patients with positive egfr status, and in patients with adenocarcinoma histology.

**Conclusions:**

For patients with advanced nsclc who progressed following first-line platinum-based chemotherapy, the data demonstrate that second-line egfr-tki treatment is efficacious and well-tolerated and that it does not appear to diminish the benefit of third-line chemotherapy.

## 1. INTRODUCTION

Lung cancer remains the leading cause of cancer-related death in both women and men in Canada and throughout the world 1,2. The estimated 24,000 Canadians who will be diagnosed with lung cancer in 2008 face a 5-year relative survival rate of 15% 1. Current survival rates reflect modest advances in anticancer therapies, considering the 5% average 5-year survival rate of the 1960s.

Non-small-cell lung cancer (nsclc) is the most common and deadly form of lung cancer 3. The current standard of care for patients with locally advanced or metastatic nsclc and adequate performance status (ps) involves up to 3 lines of systemic therapy. Platinum-based combination chemotherapy is recognized as the first-line standard of care in nsclc^4^. Upon failure of first-line therapy, 3 treatment options currently exist for second- and subsequent-line therapy (Table I). Docetaxel (Taxotere: Sanofi-Aventis Canada, Laval, QC) was the first cytotoxic agent approved for the second-line treatment of advanced nsclc based on phase iii trials showing a longer survival than that seen with with best supportive care (bsc)^6^. Erlotinib (Tarceva: Hoffmann–La Roche Ltd., Mississauga, ON), an epidermal growth factor receptor tyrosine kinase inhibitor (egfr-tki), was approved in Canada in 2005 as monotherapy for the treatment of patients with metastatic nsclc after failure of at least 1 prior chemotherapy regimen 11. Erlotinib demonstrated survival improvement in addition to control of the most distressing lung cancer symptoms and improvement in quality of life (qol). The phase iii jmei trial 7 (*n* = 571) demonstrated that the clinical efficacy of pemetrexed (Alimta: Eli Lilly Canada, Toronto, ON) was equivalent to that of docetaxel, therefore making pemetrexed a third option for therapy in recurrent nsclc.

Epidermal growth factor receptor is overexpressed in approximately 80% of nsclc cases, making it an ideal therapeutic target 12. As an egfr-tki, erlotinib competes with atp for the catalytic binding site of the egfr tyrosine kinase inside tumour cells, thus inhibiting egfr autophosphorylation and subsequent downstream signalling^13^. Agents that target egfr spare healthy cells by selectively inhibiting specific pathways involved in tumour growth and progression, resulting in a favourable safety profile as compared with chemotherapeutic agents 14.

Several trials have demonstrated the activity of egfr-tkis in recurrent nsclc. The phase iii National Cancer Institute of Canada br.21 trial 8 (*n* = 731) compared erlotinib with bsc in patients with advanced nsclc who had received 1 or 2 regimens of chemotherapy and who were not eligible for further chemotherapy. Half of the patients in the trial were treated with erlotinib as second-line therapy, and half received erlotinib as third-line therapy. As compared with bsc, treatment with erlotinib resulted in significantly longer overall survival [6.7 months vs. 4.7 months; hazard ratio (hr): 0.70; *p* < 0.001] and significantly higher progression-free survival favouring erlotinib (2.2 months vs. 1.8 months; hr: 0.6; *p* < 0.001). Patients who received erlotinib also demonstrated significantly longer stability of their lung cancer-related symptoms (dyspnea, pain, and cough), superior qol, and improved physical function as compared with patients in the placebo arm.

The subgroups with a greater likelihood of a response to erlotinib have been widely catalogued, but a multivariate analysis revealed that a non-smoking history was the only significant independent predictor of a survival effect with erlotinib 15. Never smokers receiving erlotinib had a significantly higher survival rate than did patients in the placebo arm (hr: 0.4; *p* < 0.01).

Docetaxel, erlotinib, and pemetrexed have significantly changed the treatment landscape for advanced nsclc, but the optimal selection of second-line (and subsequent third-line) therapy has yet to be firmly established. A variety of factors, such as the divergent toxicity profiles of the agents and the lack of well-defined prognostic patient characteristics, combine to confound treatment decisions in recurrent nsclc. Following first-line trials that showed no increased efficacy of concomitant egfr-tki and chemotherapy treatment over chemotherapy alone 16–19, it was proposed that these two types of agents are antagonistic when given concurrently; specifically, egfr-tkis may interfere with the cell cycle–specific cytotoxicity of chemotherapy agents 20,21.

These data led to some debate over the effectiveness of third-line chemotherapy following second-line egfr-tki treatment, but studies have demonstrated that chemotherapy can indeed be used effectively when preceded by egfr-tkis. For example, the recent trial multinational interest^22^ (*n* = 1316) demonstrated that second-line therapy with gefitinib in an unselected population provided a survival improvement similar to that of second-line chemotherapy, with a significant number of patients going on to successfully receive third-line chemotherapy. In a phase ii study of erlotinib in untreated patients, Giaccone *et al.* also reported that most patients went on to receive subsequent treatment with chemotherapy 23.

Given the similar efficacies of docetaxel, erlotinib, and pemetrexed, the incurable nature of advanced nsclc, and the clinically relevant survival seen in the second-line setting, clinicians must weigh a variety of factors when selecting second- and third-line treatment. The purpose of this retrospective practice review was to examine the outcomes of second-line erlotinib treatment followed by third-line chemotherapy in the treatment of recurrent metastatic nsclc in Canadian clinical practice.

## 2. METHODS

For this retrospective chart review, we identified patients with recurrent nsclc who had received second-line erlotinib therapy followed by third-line chemotherapy. Eligible patients were selected by census from three major Canadian cancer treatment centres. All patients had failed prior systemic cytotoxic therapy and therefore detailed first-line data are not included in this analysis.

Clinicopathologic demographics collected from patient medical records were age, ethnicity, sex, nsclc pathology (adenocarcinoma, bronchioalveolar, squamous, large-cell, nsclc not otherwise specified), tumour egfr status (positive, negative, unknown), smoking history (current/ever smoker, never smoker), and ps [Eastern Cooperative Oncology Group (ecog) 0–3] 24. First-, second-, and third-line treatment data collected included the type of treatment, reason for selecting the agent, time to progression (ttp), best response [progressive disease (pd), stable disease (sd), partial response (pr), complete response (cr)], number of cycles delivered, dose reductions and delays, reason for discontinuation, dose intensity, cancer-related hospitalizations, and date of death. The presence, onset, grade, and resolution of rash during erlotinib treatment were also recorded. Criteria used for classifying response and progression included the Response Evaluation Criteria in Solid Tumors 25, radiographs, and computed tomography or bone scans.

The study was approved by the ethics committees and health records departments at all institutions involved. Because the study was an observational, retrospective case series, power estimation and analyses for statistical significance were not performed. Demographic, treatment, and outcome data are summarized.

## 3. RESULTS

We identified 35 patients with advanced nsclc for this retrospective practice review. Selected study subjects received second-line erlotinib therapy followed by third-line chemotherapy in the period between March 2001 and April 2008. Patient data was collected from three Canadian institutions: the BC Cancer Agency, Vancouver, British Columbia (*n* = 16); the Jewish General Hospital, Montreal, Quebec (*n* = 16); and The Moncton Hospital, Moncton, New Brunswick (*n* = 3).

The study population was evenly split between the sexes and had a median age of 59 years. Most of the patients were white (66%), but Asian patients accounted for 31% of the study population. The most commonly reported pathology was adenocarcinoma (72%), and most of the patients had an ecog performance status of 1 (69%) at the time of metastatic diagnosis. The proportion of current/ever smokers closely matched the proportion of never smokers in this population. Table II presents baseline patient demographics.

### 3.1 First-Line Therapy

Of the 35 study patients, 97% received standard platinum-doublet first-line therapy for advanced nsclc. First-line regimens varied by centre; the most common doublets were cisplatin–gemcitabine (26% of patients), carboplatin–paclitaxel (23%), and carboplatin–gemcitabine (20%). The calculated mean first-line ttp of 6.6 months and 46% overall response rate (1 cr and 15 prs) suggest that the study population benefited as expected from treatment in this setting.

### 3.2 Second-Line Erlotinib Therapy

All 35 patients received second-line erlotinib treatment after failing or being unable to tolerate first-line chemotherapy. Patients were initiated on the standard dose of 150 mg once daily. In 89% of the patients, no dose reductions or delays were required throughout second-line treatment. Patients requiring dose reductions (*n* = 3) maintained a median dose intensity of 87%. The average duration of erlotinib treatment was 9.2 months and the median ttp was 7 months. Based on best response in the second line, 40% of patients experienced a pr, 26% had sd, and 34% had pd. Table III summarizes ttp results for second- and third-line therapy.

### 3.3 Third-Line Therapy

Of the 35 patients who received second-line erlotinib, 34 went on to receive third-line single-agent chemotherapy, and 1 went on to receive the egfr-tki gefitinib. The most common third-line agent was docetaxel (53%), followed by pemetrexed (24%), gemcitabine (12%), and vinorelbine (9%). According to records of best response during third-line therapy, 18% of patients exhibited a pr, 50% maintained sd, and 32% experienced pd. As expected, the mean third-line ttp of 4.3 months was the lowest of all lines of therapy. Table III compares key efficacy parameters for second- and third-line therapy, including ttp, best response, and ps before therapy.

### 3.4 Populations of Interest

**FIGURE 1:**
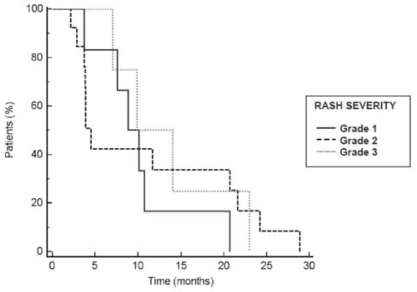
Kaplan–Meier estimate of time to progression by grade of rash

Further analysis of patient data identified populations of interest that demonstrated improved ttp with second- line erlotinib therapy. First, improved outcomes were observed in the two thirds of patients who developed rash subsequent to erlotinib treatment. The mean ttp was 10.5 months for patients with rash as compared with 5.8 months in patients without rash. Patients who developed rash were also much more likely to experience a pr to second-line erlotinib treatment (54%) than were those without rash (9%). In addition, increasing ttp was positively associated with increasing grade of rash severity. Table IV summarizes outcomes in patients who developed a rash as compared with patients who did not. These results are consistent with larger controlled trials of erlotinib for nsclc that have demonstrated a positive correlation between rash and response or survival 16,19,26,27. Figure 1 shows the Kaplan–Meier estimate of time to progression by grade of rash. All patients in this practice review that benefited beyond 10 months developed rash secondary to erlotinib treatment. All rashes were mild-to-moderate in severity. Treatment varied across centres; however, all rashes resolved completely.

Never smokers also demonstrated improved outcomes with second-line erlotinib. The mean ttp of never smokers was 12.3 months, as compared with 5.6 months for current/ever smokers. Never smokers were also much more likely to experience a pr to second-line erlotinib treatment (63%) than were current/ever smokers (13%). Table V summarizes patient outcomes according to smoking history. These results are consistent with larger controlled trials of erlotinib in advanced nsclc. An exploratory multivariate analysis of the br.21 study revealed that a history of never smoking was predictive both of response to erlotinib and of survival (hr: 0.8; *p* = 0.004) 8.

**FIGURE 2:**
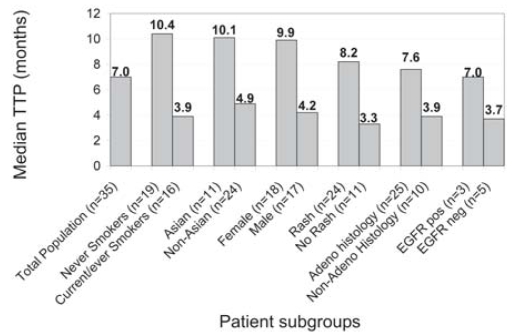
Median second-line time to progression by patient subgroup. Adeno = adenocarcinoma; egfr = epidermal growth factor receptor; pos = positive mutational status; neg = negative mutational status.

Further analyses of this study population demonstrated that female sex and Asian race also conferred improvements in ttp with second-line erlotinib therapy as compared with male sex and non-Asian race respectively. However, it should be noted that the current/ever smoker, male, and non-Asian subgroups also did better in this review than in previous reports. Our findings align with analyses from controlled trials, such as the br.21 trial, demonstrating that all patient subgroups benefit from erlotinib treatment, and that patients with certain defined clinical characteristics may do better than the average seen for all second-line agents 8. We also calculated and compared TTP for EGFR-positive and EGFR-negative patients; however, mutational status was unknown for 77% of the study population. Table VI summarizes ttp with second-line erlotinib therapy in populations of interest. Figure 2 compares median second-line ttp values for the populations of interest mentioned here.

## 4. DISCUSSION

The small size and retrospective nature of this case series limits the findings of this analysis of second-line erlotinib in nsclc patients. In 50% of cases, investigators cited clinical characteristics associated with increased response (for example, female sex, Asian race, positive egfr mutational status, non-smoking history) as the reason for prescribing erlotinib in the second-line setting. This patient selection bias is reflected in the overall patient demographics: more than half the patients enrolled were women, 72% had adenocarcinoma, 31% were Asian, and 54% were never smokers. This patient selection bias may account for the high ttp and response rate observed in this analysis. However, the median age of 59 years in this study is similar to the median age in the pivotal phase iii second- and subsequent-line advanced nsclc trials (61, 58, and 62 years respectively) 6,12,15. The proportion of patients with ps 0 or 1 in this study (73%) is also comparable to the large pivotal trials (76%, 88%, and 66% respectively) 6,7,8.

The mean first-line ttp of 6.6 months and response rate of 46% (15 pr; 1 cr) with platinum-doublet therapy suggests that the study population benefited as expected (or better) in this setting as compared with previous trials. Investigators indicated most often that chemotherapy was discontinued because of completion of therapy (49%), no response or progression (17%), and because of toxicity such as febrile neutropenia, thrombocytopenia, deep vein thrombosis, fatigue, and peripheral neuropathology (17%).

In the second-line setting, the study population demonstrated benefit from second-line erlotinib treatment in advanced nsclc. The median overall ttp of 7.0 months and the response rate of 40% with second-line erlotinib treatment in this study were greater than those reported in the pivotal phase iii studies examining outcomes in the second-line treatment of advanced nsclc 6,7,8. In our analysis, dose intensity was well maintained throughout second-line treatment, with only 3 patients requiring dose reductions. Erlotinib was well tolerated, with an average duration of treatment of 9.2 months. Investigators cited progression as the rationale for 88% of cases in which erlotinib was discontinued. Side effects, including gastrointestinal symptoms, were the reason for 12% of erlotinib discontinuations. In 5 patients, hospitalization occurred for cancer-related reasons. Clinicians noted symptom improvement in 23% of patients during second-line erlotinib treatment. In 13% of patients, ps improved; it remained stable in 50%; and it worsened in 37% of patients.

Third-line chemotherapy in this case series involved many different agents, including docetaxel (most commonly used), pemetrexed, gemcitabine, and vinorelbine. Third-line therapy following erlotinib was observed to be effective in this analysis, with a median ttp of 3.5 months and an overall response rate of 18%. These results compare closely to third-line results observed by Ng *et al.* in a similar Canadian retrospective review 28 (*n* = 21): for docetaxel, median ttp was 4.2 months with a 5% response rate.

Median time to death after discontinuation of third-line chemotherapy in the present case series was 5.4 months. Four patients are still receiving third-line chemotherapy, and 2 patients are currently receiving fourth-line therapy with docetaxel.

Our data demonstrate that metastatic nsclc patients who are able to receive third-line chemotherapy following second-line erlotinib both tolerate it and benefit from it. In the present study, we observed greater response rates and ttp from second-line therapy than has previously been reported in controlled trials. That result may be somewhat an effect of the partially selected population. Another possible explanation for the extended benefit may be clinicians’ improved ability to manage patients who develop a skin rash, and thus avoid dose reductions or discontinuations, especially in light of continuing evidence that rash is an indicator of additional benefit to the patient 29,30.

The results of our case series further support the evidence that it is appropriate to consider 3 lines of treatment in patients with advanced nsclc. In addition, erlotinib benefits patients in all demographics, and second-line egfr-tki followed by chemotherapy is an effective treatment option.

## 5. CONCLUSIONS

For patients with advanced nsclc who progress following first-line platinum-based chemotherapy, our retrospective practice review demonstrates that second-line egfr-tki treatment with erlotinib is efficacious and well tolerated. In addition, second-line erlotinib treatment does not appear to diminish benefit from subsequent third-line chemotherapy. And although certain patient subgroups exhibited improved ttp during second-line therapy, patients without the clinical characteristics associated with increased response to egfr-tkis also demonstrated benefit from erlotinib treatment.

Three lines of therapy should be considered for patients with metastatic nsclc. Choice of second-line and subsequent therapy should be individualized based on numerous considerations such as symptom improvement, ps, patient comorbidities, toxicity, qol considerations, patient preference, convenience, and ease of administration.

## Figures and Tables

**TABLE I:** Results of selected randomized phase iii trials in the second-line treatment of non-small-cell lung cancer

Reference	Agents	Patients (*n*)	Arm	Median cycles (*n*)	Response rate (%)	Median survival (mo.)	1-Year survival (%)	2-Year survival (%)	Median survival (mo.) for ps 0 or 1 in second line
Fossella *et al.*, 2000 5 (tax320)	Docetaxel compared with vinorelbine or ifosfamide (v/i)	373	D100	3	10.8	5.5	21		
			D75	3	6.7	5.7	32	na	na
			v/i	3/2	0.8	5.6	19		
Shepherd *et al.*, 2000^6^ (tax317)	Docetaxel compared with best supportive care (bsc)	104	D100	2	7.1	5.9	19		na
			D75	4	7.1	7.5	37	na	7.9
			bsc	—	—	4.6	11		6.3
Hanna *et al.*, 2004 7 (jmei)	Docetaxel compared with pemetrexed	571	D75	4	8.8	7.9	29.7	0	9.1
			Pem	4	9.1	8.3	29.7	0	9.4
Shepherd *et al.*, 2005 8 (br.21)	Erlotinib compared with bsc	731	E150	— a	8.9	6.7	31	13 b	9.4
			bsc	—	—	4.7	21	0	6.7

a Erlotinib was given until disease progression or unacceptable toxicity.

b Personal communication to author.

Adapted from Stinchcombe and Socinski, 2008 9 and Ramalingam and Sandler, 2006 10. ps = performance status; D100 = docetaxel 100 mg/m^2^ every 3 weeks; D75 = docetaxel 75 mg/m^2^ every 3 weeks; na = not available; Pem = pemetrexed 500 mg/m^2^ every 3 weeks; E150 = erlotinib 150 mg daily.

**TABLE II:** Baseline patient (*n* = 35) characteristics

*Characteristic*	n	*%*
Age (years)		
Median	59	—
Range	38–80	—
Sex		
Male	17	49
Female	18	51
Race		
Asian	11	31
White	23	66
Other	1	3
egfr status by mutation		
Positive	3	9
Negative	5	14
Unknown	27	77
Performance status a		
0	5	14
1	24	69
2	6	17
Pathology subtype		
Adenocarcinoma	25	72
Squamous cell carcinoma	4	11
Bronchioloalveolar carcinoma	1	3
Large-cell	1	3
Non-small-cell lung cancer/ not otherwise specified	4	11
Smoking status		
Current/ever smoker	16	46
Never smoker	19	54

a Eastern Cooperative Oncology Group performance status at metastatic diagnosis.

egfr = epidermal growth factor receptor.

**TABLE III:** Efficacy results for second- and third-line therapy

	Second line		Third line	
ttp (months)	*n=*33		*n=*18	
Mean	9.2		4.3	
Median	7.0		3.5	
Range	1.3–28.9		1.2–11.8	
	
	n	*%*	n	*%*
	
Best response	*n=*35		*n=*28	
Partial response	14	40	5	18
Stable disease	9	26	14	50
Progressive disease	12	34	9	32
ps before therapy	*n=*35		*n=*35	
0	9	26	7	20
1	18	51	10	28
2	7	20	17	49
3	1	3	1	3

ttp = time to progression; ps = performance status.

**TABLE IV:** Effect of rash on outcomes with second-line erlotinib therapy

	Rash (*n*=24; 67%)		No rash (*n* =11; 33%)	
ttp (months)				
Mean	10.5		5.8	
Median	8.2		3.3	
Range	2.1–28.9		1.3–13.7	
	
	n	*%*	n	*%*
	
Best response				
Partial response	13	54	1	9
Stable disease	6	25	3	27
Progressive disease	5	21	7	64
	
	*Patients (*n*)*	*Meanttp(months)*		*Medianttp(months)*
	
Grade of rash a				
1	7	9.1		8.8
2	13	10.4		3.9
3	4	13.5		12.0

a Grade 1 rash = mild; Grade 2 = moderate; Grade 3 = severe/ intolerable.

ttp = time to progression.

**TABLE V:** Smoking history and second-line erlotinib therapy

	Never smokers (*n*=19, 54%)		Current/ever smokers (*n*=16, 46%)	
ttp (months)				
Mean	12.3		5.6	
Median	10.4		3.9	
Range	2.1–28.9		1.3–14.0	
	
	*n*	%	*n*	%
	
Best response				
Partial response	12	63	2	13
Stable disease	5	26	4	25
Progressive disease	2	11	10	63

ttp = time to progression.

**TABLE VI:** Time to progression (ttp) in populations of interest with second-line erlotinib therapy

Characteristic	Patients (*n*)	ttp (months)	
		Mean	Median
Total population	35	9.2	7.0
Never smokers	19	12.3	10.4
Current/ever smokers	16	5.6	3.9
Women	18	11.1	9.9
Men	17	7.3	4.2
Asian	11	10.2	10.1
Non-Asian	24	8.8	4.9
Rash	24	10.5	8.2
No rash	11	5.8	3.3
egfr-positive	3	10.8	7.0
egfr-negative	5	8.7	3.7
Adenocarcinoma histology	25	9.4	7.6
Non-adenocarcinoma histology	10	8.9	3.9

egfr = epidermal growth factor receptor.
